# Supplementary material for: Fluorine-Containing, Self-Assembled Graft Copolymer for Tuning the Hydrophilicity and Antifouling Properties of PVDF Ultrafiltration Membranes
Source: Polymers (Basel). 2023 Sep 1;15(17):3623. doi: 10.3390/polym15173623 (PMC10490059; doi:10.3390/polym15173623)
Supplement: Supplementary file 1 [file polymers-15-03623-s001.zip › polymers-2583586-supplementary.pdf]

Supporting Information

Fluorine-Containing, Self-Assembled Graft Copolymer for Tuning the Hydrophilicity and Antifouling Properties of PVDF Ultrafiltration Membranes

Seung Jae Moon, Young Jun Kim, Du Ru Kang, So Youn Lee and Jong Hak Kim*

Department of Chemical and Biomolecular Engineering, Yonsei University,

50 Yonsei-ro, Seodaemun-gu, Seoul 03722, South Korea

*Correspondence should be addressed to:

E-mail: jonghak@yonsei.ac.kr

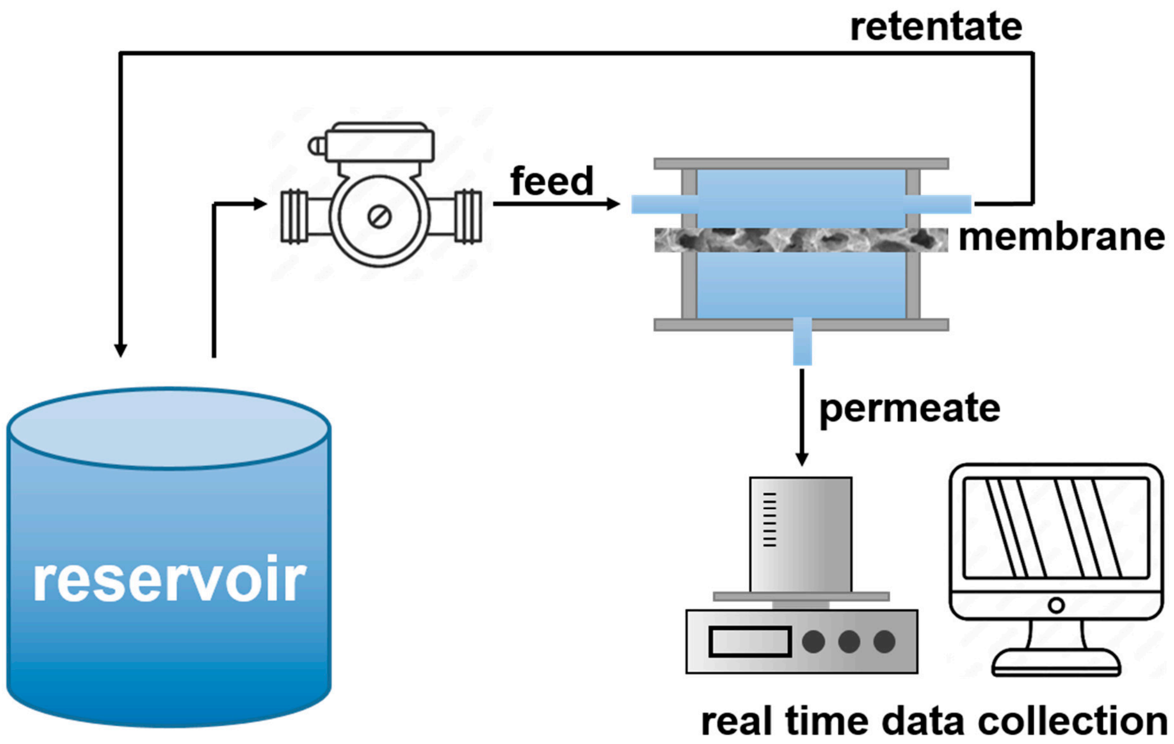

Figure S1. Schematic diagram of the setup for water permeation test.

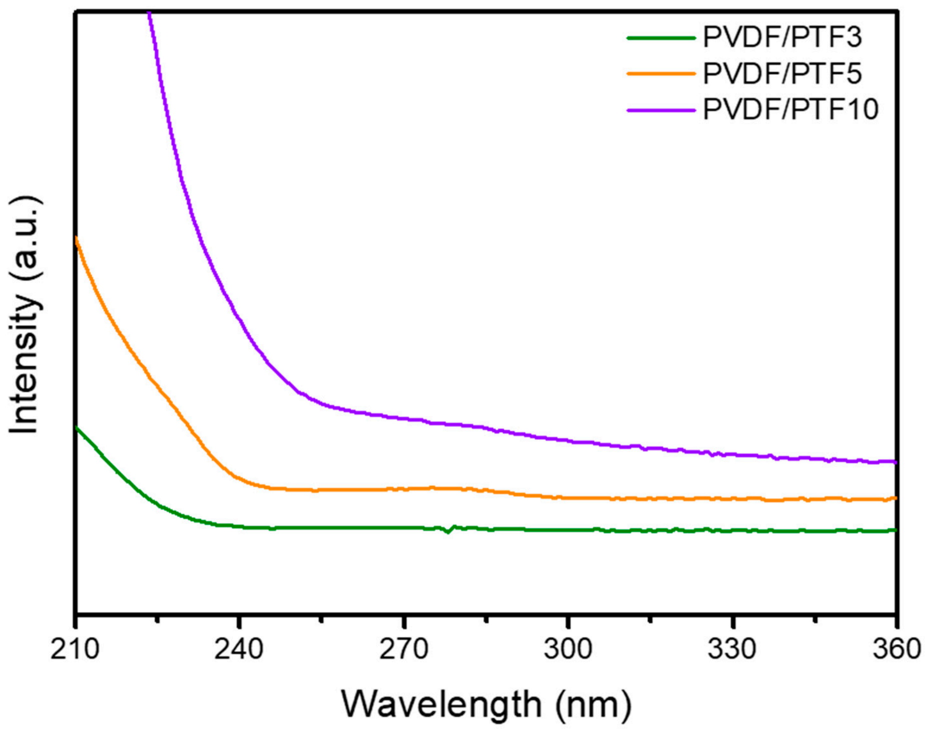

Figure S2. UV-vis spectra of BSA solutions obtained after filtration test through PVDF/PTF membranes.

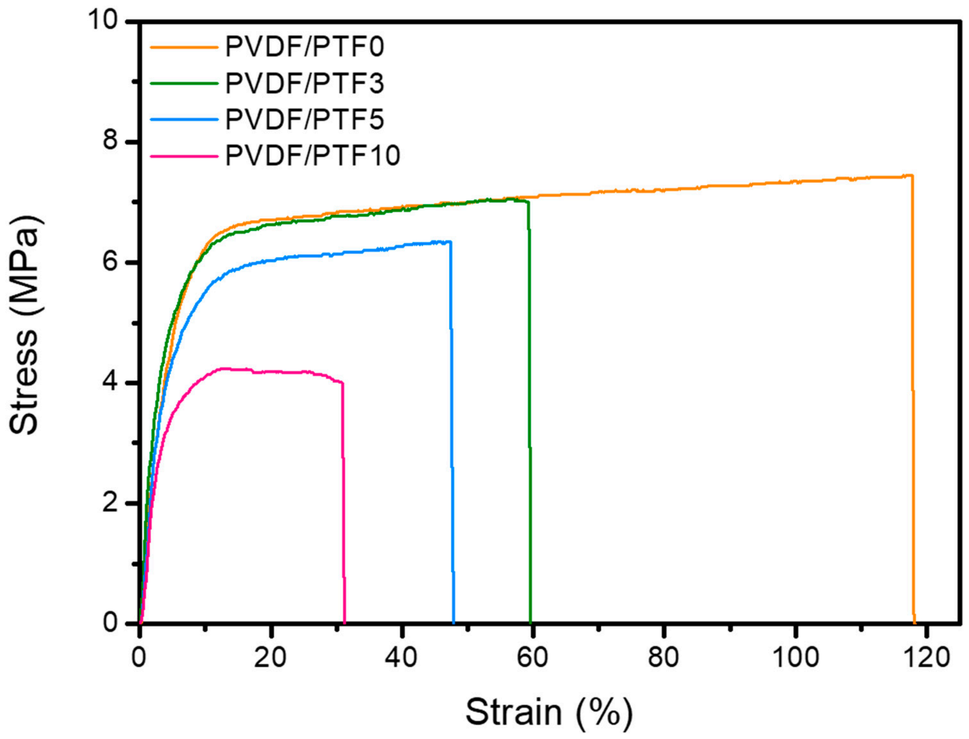

Figure S3. Stress–strain curves of PVDF/PTF membranes with various PTF loadings.

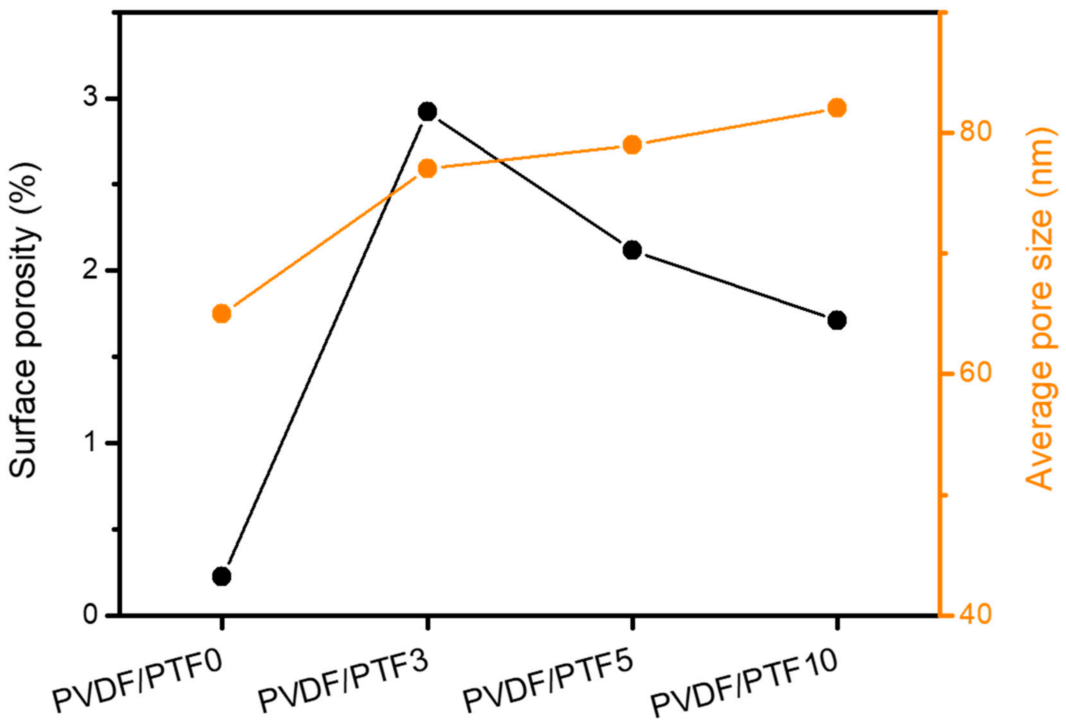

Figure S4. Surface porosity and average pore size of PVDF/PTF membranes with various PTF loadings.

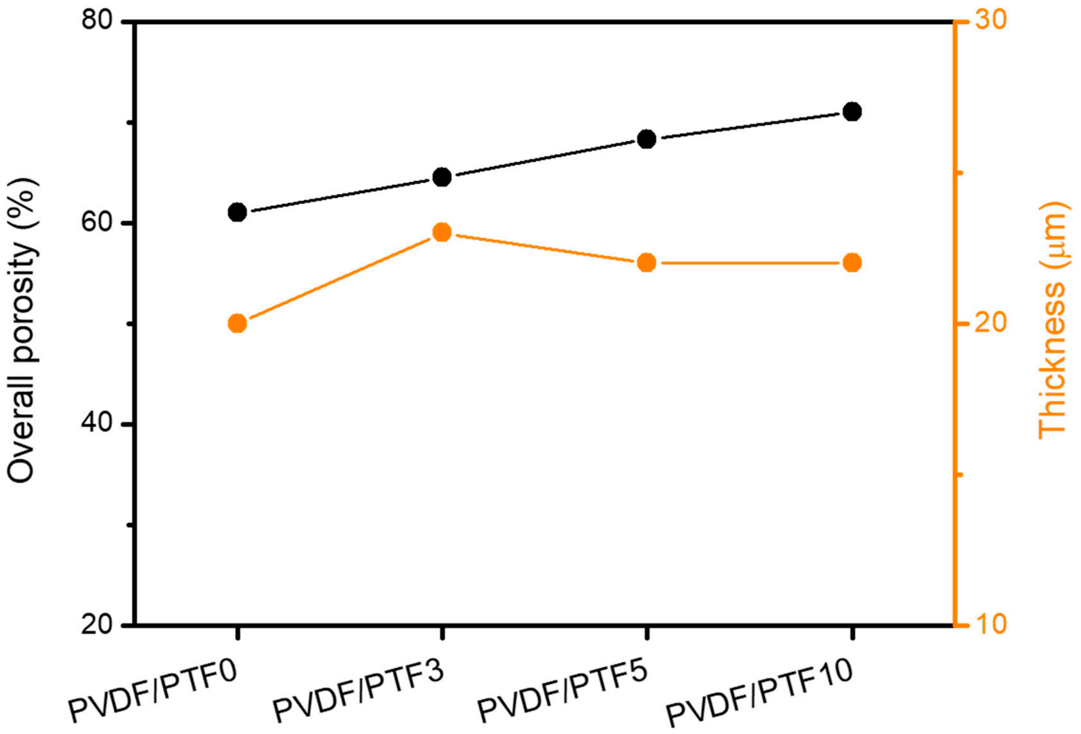

Figure S5. Overall porosity and thickness of PVDF/PTF membranes with various PTF loadings.
